# Draft Genome Sequence of Kitasatospora griseola Strain MF730-N6, a Bafilomycin, Terpentecin, and Satosporin Producer

**DOI:** 10.1128/genomeA.00208-15

**Published:** 2015-03-26

**Authors:** Jennifer C. Arens, Brad Haltli, Russell G. Kerr

**Affiliations:** a Department of Biomedical Sciences, University of Prince Edward Island, Charlottetown, Prince Edward Island, Canada; b Department of Chemistry, University of Prince Edward Island, Charlottetown, Prince Edward Island, Canada

## Abstract

We report here the draft genome sequence of Kitasatospora griseola strain MF730-N6, a known producer of bafilomycin, terpentecin, and satosporins. The current assembly comprises 8 contigs covering 7.97 Mb. Genome annotation revealed 7,225 protein coding sequences, 100 tRNAs, 40 rRNA genes, and 23 secondary metabolite biosynthetic gene clusters.

## GENOME ANNOUNCEMENT

The presence of numerous secondary metabolite gene clusters within a single genome is a common feature in actinomycetes and has resulted in the sequencing of genomes from numerous organisms within this order, including streptomycetes (1) and the closely related kitasatosporae (2). Four kitasatosporae genome sequences are currently published (3–5), and an additional 8 genomes are in various phases of completion (JOGH00000000.1, JQMO00000000.1, JQLN00000000.1. JNYV00000000.1, JNWZ00000000.1, JNYE00000000.1, JNYQ00000000.1, and JNWY00000000.1). Given the large number of secondary metabolite biosynthetic gene clusters within their genomes and the relative lack of genome data in comparison to other actinomycete genera, there is a possibility of discovering new natural products with therapeutic potential from the underexplored genomes of kitasatosporae.

K. griseola strain MF730-N6 was isolated from Japanese soil and is responsible for the production of the diterpene terpentecin (6) and polyketides belonging to the bafilomycin (7) and satosporin (8) families. K. griseola MF730-N6 was obtained from the International Patent Organism Depositary (Nashihara, Japan) under the accession number FERM BP-1045. Genomic DNA was isolated using the Qiagen genomic tip 100/G kit. Library preparation (long insert ~20 kb) and sequencing using the Pacific Biosciences RS II platform was performed by the McGill University and Génome Québec Innovation Centre. Sequencing of 8 SMRT cells generated 1,377,430 subreads (2,920,331,552 bases) and 77,677 circular consensus sequences (175,515,662 bases), resulting in 324- and 18-fold coverage, respectively (assuming a 9-Mb genome). *De novo* assembly of corrected reads was performed by the sequencing facility using the hierarchical genome assembly process 2 analysis pipeline (9). The assembly consisted of 15 contigs; however, closure of 5 gaps was achieved by performing a *de novo* assembly using the Geneious assembler version 7.0.6 and sequencing amplicons spanning overlapping regions. The resulting assembly contained 8 contigs ranging in size from 5.9 kb to 3.5 Mb with a contig *N*_50_ of 2,590,787 bp.

The K. griseola draft genome comprised 7,966,157 bp with an overall G+C content of 72.7%. The genome was annotated using the RAST server (10) and the NCBI Prokaryotic Genome Automatic Annotation Pipeline (NCBI annotation submitted to GenBank). Annotation identified a total of 7,225 protein-coding sequences, 100 tRNA genes, and 40 rRNA genes forming 9 complete and 5 incomplete rRNA operons. Orthologs of almost all developmental regulatory genes (11), with the exception of *bldB* and *whiJ*, as well as orthologs of *ram* cluster genes involved in aerial mycelia formation, were located within the K. griseola genome (12).

AntiSMASH analysis of the K. griseola MF730-N6 genome revealed 23 putative secondary metabolite biosynthetic gene clusters (13). Although gene clusters for known metabolites, such as hopanoids, germacradienol/geosmin, bafilomycin, spore pigment, a valanimycin-like compound, terpentecin, satosporin, and a spore-associated protein, were identified within the genome, 15 of the 23 gene clusters had unknown products; the isolation of other putatively novel natural products from this organism is thus promising and will be the subject of future studies.

### Nucleotide sequence accession numbers.

This whole-genome shotgun project has been deposited at DDBJ/EMBL/GenBank under the accession number JXZB00000000. The version described in this paper is version JXZB01000000.
